# Patient Assessment of Chronic Illness Care, Glycemic Control and the Utilization of Community Health Care among the Patients with Type 2 Diabetes in Shanghai, China

**DOI:** 10.1371/journal.pone.0073010

**Published:** 2013-09-10

**Authors:** Li-Juan Liu, Yun Li, Kun Sha, Yue Wang, Xiang He

**Affiliations:** 1 Office of Medical Education, Training Department, the Second Military Medical University, Shanghai, People's Republic of China; 2 Department of Cell and Developmental Biology, University of Illinois, Urbana-Champaign, Urbana, Illinois, United States of America; 3 Training Department, the Second Military Medical University, Shanghai, People's Republic of China; Iran University of Medical Sciences, (Islamic Republic of Iran)

## Abstract

**Objectives:**

To evaluate the relationship between Patient Assessment of Chronic Illness Care in community health centers and self-management behaviors and glycemic control and to examine the relationship between Patient Assessment of Chronic Illness Care in community health centers and the utilization of community health centers for monitoring and treating diabetes among the patients with type 2 diabetes.

**Methods:**

A questionnaire including self-management behaviors, glycemic control, Patient Assessment of Chronic Illness Care in community health centers and the most often utilized medical institutions for monitoring and treating diabetes (community health centers vs. hospitals) was administered to 960 patients with type 2 diabetes in Shanghai, China. The relationships between Patient Assessment of Chronic Illness Care and self-management behaviors, self-management behaviors and glycemic control, Patient Assessment of Chronic Illness Care and glycemic control, Patient Assessment of Chronic Illness Care and the most often utilized medical institutions for monitoring and treating diabetes were examined.

**Results:**

Wilcoxon rank sum tests showed that the high scores of total Patient Assessment of Chronic Illness Care and five subscales in community health centers were positively related to almost all the proper self-management behaviors and good glycemic control (*p*<0.05). Almost all of the proper self-management behaviors were positively related to good glycemic control (*p*<0.01). High summary score of the Patient Assessment of Chronic Illness Care was positively associated with the utilization of community health centers for monitoring and treating diabetes (*p*<0.001).

**Conclusions:**

Patient Assessment of Chronic Illness Care (implementation of the Chronic Care Model) in community health centers was associated with patients' self-management behaviors and glycemic control, and finally was associated with the utilization of community health centers for monitoring and treating diabetes.

## Introduction

As the elderly population continues to grow in China, the prevalence of chronic diseases is also on the rise. Chronic diseases continue to be a significant burden on the health care system in China. In China, Shanghai has the heaviest burden of chronic diseases, because it has the largest population and the largest ageing population [1]. Over 4.5 million people in Shanghai suffer from chronic health problems, and this number is increasing [2]. Diabetes mellitus is one of the major chronic diseases among Chinese adults, which causes a significant health care burden due to the associated complications.

Chronic care, its management, and quality are themes being addressed worldwide [3]. Prevention and management of chronic disease is an urgent primary health problem to be addressed in Shanghai. Efforts to improve the quality of chronic disease management in community health centers have been made in Shanghai. Community health centers were mainly responsible for primary care and preventive health care of chronic diseases, including implementing electronic registry, establishing electronic patient records, providing primary care, conducting health education for patients and following-up patients, which were mainly provided by general practitioners and preventive health experts. Hospitals were mainly responsible for specialized treatment of chronic diseases, which were mainly provided by specialists. There was continuity of care in community health centers, but not in hospitals. The costs of chronic care in community health centers were usually much lower than that in hospitals, but many patients believe that the quality of services provided by community health centers was lower than that provided by hospitals in China [4]–[5]. Therefore, the overuse of high level hospitals and the underuse of primary care services by patients have been widespread in China [6].

High quality of chronic care had positive effects on the formation of proper self-management behaviors. A previous study had shown the relationship between the Patient Assessment of Chronic Illness Care (PACIC) and increased exercise in patients with diabetes [7]. Proper self-management of diabetes was important to glycemic control. We hypothesized that high PACIC in community health centers was positively related to patients' proper self-management behaviors and good glycemic control, which could increase the patients' trust in community health centers. The more the patients trusted in community health centers, the more they would be likely to use community health centers, which would be helpful to reduce the costs of chronic care.

Although a previous study has addressed the relationship between total PACIC and self-management behaviors [8] among the diabetic patients, few studies have evaluated the relationship between PACIC in community health centers and glycemic control and the relationship between PACIC in community health centers and the utilization of community health centers for monitoring and treating diabetes in a general representative Chinese diabetic population.

This study was carried out using the patients with type 2 diabetes as subjects with four main objectives: to examine the relationship between the PACIC in community health centers and the patients' self-management behaviors, to examine the relationship between the patients' self-management behaviors and glycemic control, to examine the relationship between the PACIC in community health centers and glycemic control, and to examine the relationship between the PACIC in community health centers and the utilization of community health centers for monitoring and treating diabetes.

## Materials and Methods

### Subjects

The Centers for Disease Control and Prevention in Shanghai had developed a health registration system for local residents and subsequently implemented a management system for diabetic patients. Community health centers were responsible for the management of chronic diseases. Every community health center had a health registration system for the local residents with diabetes within its range of services and subsequently implemented health management for diabetic patients. The target population of this study was the patients who had been diagnosed type 2 diabetes in Yangpu and Baoshan districts. We randomly chose 6 community health centers (by different economic status, three community health centers each in Yangpu and Baoshan). Two hundred patients who had been diagnosed type 2 diabetes were randomly selected from each community health center. A total of 1200 patients who had been diagnosed type 2 diabetes were invited to participate in the study. The questionnaire was administered in the patients' home. Of all the participants, 960 participants completed the questionnaire thoroughly. The response rate of effective questionnaires was 80.0% (960/1200).

### Ethics Statement

Written informed consents were obtained from each participant. This study was approved by the Biological and Medical Ethics Committee, the Second Military Medical University.

### Instruments

PACIC was developed to assess congruency of provided health care to the Chronic Care Model (CCM) [9]. Respondents rated how often they experienced the content described in each item during the past 6 months. Each item was scored on a 5-point scale ranging from 1 (almost never) to 5 (almost always) [10]. There are five PACIC subscales: patient activation, delivery system/practice design, goal setting/tailoring, problem solving/contextual, and follow-up/coordination [11]. The total PACIC and five subscales were scored out of a possible total of 5 [7]. The PACIC was validated in a sample of patients with diabetes [12].

Information on subject age, gender, education (no education, primary school, junior high school, senior high school and holding a degree or diploma), marital status (currently married, single including never married, divorced, separated and widowed), income (very low: household per capita annual income<10,000RMB, low: 10,000RMB≤household per capita annual income<20,000RMB, middle: 20,000RMB≤household per capita annual income<40,000RMB, high: 40,000RMB≤household per capita annual income<100,000RMB, very high: 100,000RMB≤household per capita annual income), suffering from other chronic diseases beyond diabetes (yes, no), the most often utilized medical institutions for monitoring and treating diabetes (hospitals, community health centers), self-management behaviors including following regular exercise schedule(exercising 30 or more than 30 minutes almost every day) (yes, no), following a low-fat diet, such as vegetables, fish, lean meat, skim milk or low-fat milk, et al (always or almost always, sometimes or never), being able to maintain recommended weight (18.5≤Body Mass Index (BMI)≤23.9, BMI = weight (kg)/height(m)^2^)(yes, no), asking about medication side effects when taking a new prescription (always or almost always, sometimes or never), reading about side effects when taking new prescription medication (always or almost always, sometimes or never), taking diabetes medications as recommended (yes, no), checking feet for cracks and calluses (yes, no) were collected from subject self-report.

Community diabetes prevention and treatment guidelines in Shanghai provided glycemic control targets for diabetic patients. Diabetic control was poor if the frequency of bad results of blood glucose was more than 25% in the most recent year (bad  =  fasting blood glucose of venous plasma >7.0 mmol/L, or postprandial blood glucose of venous plasma >10.0 mmol/L, or fasting blood glucose of capillary whole blood >8.0 mmol/L, or postprandial blood glucose of capillary whole blood >11.0 mmol/L). Otherwise, diabetic control was good. The doctors in community health centers followed up the patients' blood glucose once every three months. Therefore, the patients monitored the blood glucose at least once every three months and four times a year. After the patients were told the criterions for the judgment of good and poor diabetic control, they were asked whether the diabetic control was good or poor according to the results of blood glucose in the most recent year. If the patients had difficulties in judging the quality of diabetic control, the investigators would help them to judge the quality of diabetic control according to the results of blood glucose in the most recent year.

### Data analysis

The data of total PACIC and five subscales were all skew distributions. Wilcoxon rank sum tests were used to examine the relationships between PACIC in community health centers and self-management behaviors, PACIC in community health centers and glycemic control, and PACIC in community health centers and the utilization of community health centers. Chi-square tests were used to examine the relationships between self-management behaviors and glycemic control. To eliminate confounders, a logistic regression model with backward conditional analysis was carried out using the most often utilized medical institutions (hospitals vs. community health centers) for monitoring and treating diabetes as dependent variable and age, gender, education, marital status, income, suffering from other chronic diseases beyond diabetes, and the total PACIC score as independent variables to examine the relationship between the total PACIC in community health centers and the utilization of community health centers. *P<*0.05 was considered significant. All data were analyzed with the SPSS 10.0 statistical analysis software package [13].

## Results

### Socio-demographic characteristics

Ages of these respondents ranged from 36 to 89 years (mean = 68.33±10.37 years). The socio-demographic characteristics of the patients were seen in Table 1.

**Table 1:** The characteristics of the patients with type 2 diabetes (N = 960).

*Factors*	*n*	*percent*
**Gender**		
Male	380	39.6
Female	580	60.4
**Age**		
<50 years old	35	3.6
50–59 years old	180	18.8
60–69 years old	290	30.2
70–79 years old	310	32.3
≥80 years old	145	15.1
**Educational level**		
No education	35	3.6
Primary school	165	17.2
Junior high school	220	22.9
Senior high school	165	17.2
Holding a degree or diploma	375	39.1
**Marital status**		
Married	760	79.2
Single (never married, divorced, separated and widowed)	200	20.8
**Income**		
Very low	5	0.5
Low	40	4.2
Middle	695	72.4
High	215	22.4
Very high	5	0.5

### Health status

Of them, 23.4% had diabetes only; 76.6% had diabetes in combination with other chronic diseases. Of the patients with other chronic diseases, 68.7% had one chronic condition in addition to diabetes, and 31.3% had two or more chronic conditions in addition to diabetes.

### The relationship between PACIC in community health centers and self-management behaviors

Of all the respondents, 57.3% followed regular exercise schedule, 67.7% always or almost always followed a low-fat diet, 52.1% was able to maintain recommended weight, 53.6% always or almost always asked about medication side effects when took a new prescription, 78.6% always or almost always read about side effects when took new prescription medication, 90.1% took diabetes medications as recommended, 49.5% checked feet for cracks and calluses.

Wilcoxon rank sum tests showed that the total PACIC and five subscales in community health centers were positively related to almost all the self-management behaviors among the diabetic patients (Table 2).

**Table 2:** The relationships between PACIC scores and different self-management behaviors (N = 960).

*Self-Management Behaviors*	*Patient Activation*		*Delivery System/Practice design*		*Goal Setting/Tailoring*		*Problem Solving/Contextual*		*Follow-up/Coordination*				*PACIC Summary Score*	
	*Median (interquartile range 25–75%)*	*P*	*Median (interquartile range 25–75%)*	*P*	*Median (interquartile range 25–75%)*	*P*	*Median (interquartile range 25–75%)*	*P*	*Median (interquartile range 25–75%)*			*P*	*Median (interquartile range 25–75%)*	*P*
**Follow regular exercise schedule**		<.001		<.001		.001		<.001				<.001		<.001
Yes	3.00 (2.33–4.00)		2.67 (2.00–3.33)		2.60 (2.00–3.00)		2.63 (2.00–3.75)		2.80 (1.80–3.20)				2.60 (2.05–3.40)	
No	2.50 (1.67–3.33)		2.33 (1.67–3.00)		2.40 (1.60–3.00)		2.50 (1.50–3.25)		2.10 (1.40–3.20)				2.45 (1.60–3.15)	
**Follow a low-fat diet**		<.001		<.001		.096		.001				.001		<.001
Always or almost always	3.00 (2.00–3.67)		2.67 (2.00–3.33)		2.60 (2.00–3.00)		2.75 (1.75–3.75)		2.60 (1.60–3.20)				2.68 (1.90–3.30)	
Sometimes or never	2.67 (1.67–3.33)		2.17 (1.33–3.00)		2.50 (1.80–3.20)		2.25 (1.75–3.25)		2.00 (1.40–3.20)				2.30 (1.65–2.95)	
**Beable to maintain recommended weight**		<.001		<.001		<.001		.004				<.001		<.001
Yes	3.00 (2.33–3.67)		2.67 (2.00–3.58)		2.80 (2.20–3.20)		2.63 (2.00–3.50)			2.80 (1.60–3.60)			2.75 (1.93–3.40)	
No	2.50 (2.00–3.33)		2.33 (1.67–3.00)		2.20 (1.60–3.00)		2.38 (1.50–3.69)			2.20 (1.40–3.00)			2.38 (1.65–3.04)	
**Ask about medication side effects when taking a new prescription**		<.001		<.001		<.001		<.001			<.001			<.001
Always or almost always	3.00 (2.00–4.00)		2.67 (2.00–3.33)		2.80 (2.00–3.00)		3.00 (1.75–4.00)			2.80 (1.80–3.20)			2.90 (1.90–3.40)	
Sometimes or never	2.67 (2.00–3.33)		2.33 (1.67–3.00)		2.40 (1.80–3.00)		2.50 (1.75–3.00)			2.20 (1.40–3.20)			2.40 (1.75–3.05)	
**Read about side effects when taking new prescription medication**		<.001		<.001		<.001		<.001			<.001			<.001
Always or almost always	3.00 (2.00–3.67)		2.67 (2.00–3.33)		2.80 (2.00–3.20)		2.75 (2.00–3.75)			2.80 (1.60–3.40)			2.80 (2.05–3.40)	
Sometimes or never	2.33 (1.33–2.67)		2.00 (1.67–2.67)		2.00 (1.20–2.40)		1.75 (1.25–2.50)			1.60 (1.40–2.60)			1.85 (1.45–2.55)	
**Take diabetes medications as recommended**		<.001		.001		.039		<.001			.015			<.001
Yes	3.00 (2.00–3.67)		2.67 (2.00–3.33)		2.60 (2.00–3.00)		2.50 (1.75–3.75)			2.60 (1.60–3.20)			2.60 (1.80–3.30)	
No	2.33 (1.33–3.33)		2.00 (1.33–3.00)		2.20 (1.40–3.00)		2.00 (1.50–3.00)			2.00 (1.40–3.00)			2.10 (1.50–3.00)	
**Check feet for cracks and calluses**		<.001		<.001		<.001		<.001			<.001			<.001
Yes	3.00 (2.00–3.67)		2.67 (2.00–3.33)		2.80 (2.20–3.20)		3.00 (2.00–3.75)			3.00 (2.00–3.40)			2.90 (2.05–3.50)	
No	2.67 (1.67–3.33)		2.00 (1.67–3.00)		2.40 (1.40–3.00)		2.25 (1.25–3.25)			1.80 (1.40–3.00)			2.20 (1.55–3.05)	

### The relationship between self-management behaviors and glycemic control

Of all the respondents, 82.8% had good glycemic control, and 17.2% had poor glycemic control. Chi-Square tests showed that almost all of the proper self-management behaviors were positively related to good glycemic control among the patients with type 2 diabetes (Table 3).

**Table 3:** The relationships between different self-management behaviors and glycemic control, and the relationships between PACIC and glycemic control (N = 960).

*Factors*	*Good glycemic control (% or mean± SD score)*	*Poor glycemic control (% or mean± SD score)*	*χ^2^ or z*	*p*
**Self-Management Behaviors**				
**Follow regular exercise schedule**			21.054	<.001
Yes	87.6	12.4		
No	76.3	23.7		
**Follow a low-fat diet**			63.977	<.001
Always or almost always	89.5	10.5		
Sometimes or never	68.7	31.3		
**Beable to maintain recommended weight**			61.883	<.001
Yes	92.0	8.0		
No	72.8	27.2		
**Ask about medication side effects when taking a new prescription**			3.254	.071
Always or almost always	84.9	15.1		
Sometimes or never	80.4	19.6		
**Read about side effects when taking new prescription medication**			38.609	<.001
Always or almost always	86.8	13.2		
Sometimes or never	68.3	31.7		
**Take diabetes medications as recommended**			15.342	<.001
Yes	84.4	15.6		
No	68.4	31.6		
**Check feet for cracks and calluses**			8.107	.004
Yes	86.3	13.7		
No	79.4	20.6		
**PACIC**				
Patient Activation	3.00 (2.00–3.67)	2.00 (1.67–2.67)	−9.838	<.001
Delivery System/Practice design	2.67 (2.00–3.33)	2.00 (1.33–3.00)	−6.508	<.001
Goal Setting/Tailoring	2.80 (2.00–3.20)	2.00 (1.40–2.40)	−7.222	<.001
Problem Solving/Contextual	2.75 (2.00–3.75)	1.75 (1.25–2.25)	−8.418	<.001
Follow-up/Coordination	2.80 (1.60–3.20)	1.60 (1.20–2.60)	−7.248	<.001
PACIC Summary Score	2.75 (2.00–3.35)	1.85 (1.40–2.35)	−8.773	<.001

### The relationship between PACIC in community health centers and glycemic control

Wilcoxon rank sum tests showed that the high scores of total PACIC and five subscales in community health centers were positively related to good glycemic control (Table 3).

### The relationship between PACIC in community health centers and the utilization of community health centers for monitoring and treating diabetes

Of all the respondents, 44.3% selected community health centers as the most often utilized medical institutions for monitoring and treating diabetes, and 55.7% selected hospitals as the most often utilized medical institutions for monitoring and treating diabetes. Wilcoxon rank sum tests showed that the high scores of total PACIC and five subscales in community health centers were positively related to the utilization of community health centers for monitoring and treating diabetes (Table 4). In our study, logistic regression analysis indicated that female, old age and high PACIC summary score were positively related to the utilization of community health centers for monitoring and treating diabetes and high education level and suffering from other chronic diseases beyond diabetes were negatively related to the utilization of community health centers for monitoring and treating diabetes (Table 5).

**Table 4:** The relationships between PACIC and the most often utilized medical institutions for monitoring and treating diabetes among the patients with type 2 diabetes (N = 960).

*PACIC*	*hospitals, median(interquartile range 25–75%)*	*community health centers, median (interquartile range 25–75%)*	*z*	*p*
Patient Activation	2.67 (1.67–3.67)	3.00 (2.00–3.67)	−3.335	.001
Delivery System/Practice design	2.33 (1.67–3.33)	2.67 (2.00–3.33)	−3.294	.001
Goal Setting/Tailoring	2.40 (1.40–3.00)	2.60 (2.00–3.20)	−4.433	<.001
Problem Solving/Contextual	2.25 (1.50–3.25)	2.75 (2.00–3.75)	−5.180	<.001
Follow-up/Coordination	2.00 (1.40–3.20)	2.60 (1.80–3.40)	−3.745	<.001
PACIC Summary Score	2.35 (1.60–3.20)	2.75 (2.00–3.30)	−4.841	<.001

**Table 5:** Logistic regression analysis^a^ including variables associated with the most often utilized medical institutions^b^ for monitoring and treating diabetes among the patients with type 2 diabetes (N = 960).

Variable	*B*	*Wald*	*P*	*Odds ratio*	*95% confidence interval of the odds ratio*
Gender^c^	.338	5.752	.016	1.403	1.064–1.849
Age	.025	11.224	.001	1.025	1.010–1.040
Education^e^	−.229	13.443	<.001	.796	0.704–0.899
Suffering from other chronic diseases beyond diabetes^f^	−.615	13.392	<.001	.541	0.389–0.752
PACIC Summary Score^g^	.466	32.252	<.001	1.593	1.357–1.871
Constant	−1.260	2.627	.105	.284	-
Marital status^h^	-	-	.362	-	-
Income^i^	-	-	.887	-	-

Note:

a Probability for stepwise: entry = 0.05; removal = 0.10.

b hospitals = 1; community health centers = 2.

c Men = 1; wemen = 2.

e No education = 1; primary school = 2; junior high school = 3; senior high school = 4; holding a degree or diploma = 5.

f No = 1; yes = 2.

g Scores ranged from 1 to 5, with higher scores indicating high quality of chronic care.

h Single (never married, divorced, separated and widowed) = 1; married = 2.

i Very low = 1; low  = 2; middle = 3; high = 4; very high = 5.

## Discussion

Diabetes is a major health problem affecting an increasing number of individuals. Patients with diabetes differ in the amount of effort they invest in self-care behaviors and in adherence to treatment of diabetes. A previous study had shown a relationship between PACIC and increased exercise in patients with diabetes [12]. This study found that the total PACIC and five subscales in community health centers were positively related to almost all the self-management behaviors among the diabetic patients. It was concluded that greater implementation of the CCM in community health centers might be helpful to improve patients' self-management behaviors.

The diabetic patients would benefit from the improvement of self-management behaviors. Exercise and diet had been clearly demonstrated to have benefits on glycemic control [14], [15]. The study also found that the better self-management behaviors were, the better glycemic control was among the patients with type 2 diabetes. Since high PACIC in community health centers was positively associated with proper self-management behaviors, it might be associated with glycemic control. The study found that high scores of the total PACIC and five subscales in community health centers was positively associated with good glycemic control, indicating that the great implementation of the CCM in community health centers had positive association with good glycemic control.

Glycemic control was very important for people with diabetes. Poor glycemic control would increase the risk of complications [16], which may place an additional strain on patients' quality of life.

As the burden of chronic non-communicable diseases is increasing in China, the roles of community health services for the management of patients with chronic diseases have been increasingly strengthened [17], [18]. In developed countries, community health centers are usually the first point of contact for patients. In China, many patients believe that the quality of services provided by community health centers is low [4], [5]. Therefore, compared with community health centers, the patients were more likely to use high level hospitals.

High PACIC in community health centers was positively associated with patients' proper self-management behaviors and good glycemic control, which would increase the patients' trust in community health centers. The more the patients trusted in community health centers, the more they would be likely to use community health centers. The results showed that the PACIC in community health centers was positively associated with the utilization of community health centers for monitoring and treating diabetes among the patients with type 2 diabetes, which was consistent with our hypothesis.

The covariates such as age, gender, education and suffering from other chronic diseases beyond diabetes have an independent impact on the patients' utilization of community health centers for monitoring and treating diabetes. Female and old age were positively associated with the utilization of community health centers for monitoring and treating diabetes. The female was more likely to live economically than the male. The costs in community health centers were usually much lower than that in hospitals. The relatively low expenses in community health centers had attracted female patients. Older age was often associated with health problems and irreversible decrease in function capacity [19]–[21], which decreased the accessibility to health-care services. Community health services were more accessible for older people than the services provided by hospitals. Therefore, older age was positively associated with the utilization of community health centers for monitoring and treating diabetes. High education level and suffering from other chronic diseases beyond diabetes were negatively associated with the utilization of community health centers for monitoring and treating diabetes. In China, the costs in community health centers were usually much lower than that in hospitals. Many patients believe that the quality of services provided by community health centers was lower than that provided by hospitals. Higher education might be associated with higher income. Highly educated people usually had high expectations for the quality of chronic care. Therefore, the patients with high education were more likely to use the services provided by hospitals for monitoring and treating diabetes compared with the patients with low education. The conditions of some patients suffering from other chronic diseases beyond diabetes were very complex. Compared with the community health centers, hospitals had more advantages in dealing with complex diseases. Therefore, suffering from other chronic diseases beyond diabetes was negatively associated with the utilization of community health centers for monitoring and treating diabetes.

After adjustment for possible confounders, the results suggested that high PACIC in community health centers was also positively associated with the utilization of community health centers for monitoring and treating diabetes. In China, the development of community health services was still on the early stage. To cost-effectively control diabetes and other chronic conditions, the further development and increasing use of community health centers in China was essential. To increase use of community health centers in China, the CCM should be well implemented in community health centers.

### Limitations

The major limitation is the cross-sectional design. Based on the questions about “ask about medication side effects” and “read about side effects when taking new medications”, the possibility that what we were seeing here were the “activated” patients who were more likely to score high on PACIC, have better self-management behaviors and report better glucose control could not be excluded.

## Conclusions

This study suggested that quality of diabetes care (implementation of the CCM) in community health centers was associated with patients' self-management behaviors and glycemic control, and finally was associated with the utilization of community health centers for monitoring and treating diabetes. As the burden of the chronic non-communicable diseases was increasing in China, community health service was an effective way to cost-effectively control diabetes and other chronic conditions. To increase the utilization of community health centers, it was necessary to improve the quality of chronic care (implementation of the CCM) in community health centers.
